# Supplementary material for: Assessing COVID-19 transmission through school and family networks using population-level registry data from the Netherlands
Source: Sci Rep. 2024 Dec 28;14:31248. doi: 10.1038/s41598-024-82646-7 (PMC11682366; doi:10.1038/s41598-024-82646-7)
Supplement: Supplementary file 1 — Supplementary Information. [file 41598_2024_82646_MOESM1_ESM.pdf]

A. Supplementary Information

A.1. Detailed explanation of datasets and variables used

All datasets at individual level available at Statistics Netherlands (CBS) are linkable to each other through a unique identifier (the combination of the variables *RINPERSOON* and *RINPERSOONS*, which together identify individuals in the data).

A.1.1. COVID-19 PCR tests *(*CORONIT/CoronIT_GGD_testdata_20210921) dataset

The COVID-19 dataset includes test results from PCR tests conducted outside of hospitals. Key variables include the individual's persistent identifier (*RINPERSOON*), test date (*DatumMonster-afname*), and test result (*Testuitslag*). Reinfections were uncommon during the study period, so we retained only the first infection date for each individual.

A.1.2. Person Network (*PN/PersNw2018_v1.0*) dataset

At the time of the study, the Person Network dataset compiled by CBS was only available for 2018. We used this dataset to extract family relations. This dataset includes the identifier (*RIN-PERSOON(S)*) of the two individuals and the type of connection (e.g. *103* for full-siblings, *102* for co-parents, *104* for parent-child relationships).

A.1.3. Registrations in primary school (*Onderwijs-INSCHRWPOTAB*) dataset

To assess if students attended primary education (ages 4 to 12) together, we used the dataset of student registrations in primary schools (*Onderwijs-INSCHRWPOTAB*), keeping the school category (*WPOTYPEPO*) "BO" or "Basisonderwijs" (the variable label was changed by CBS, but there was no change in the school systems). This only excludes children that require extra help in special schools—such as blind or deaf students—and whose infection patterns are likely to be different. Each school is associated to a school denomination, *WPODENOMINATIE*. The school denomination denotes the type of school and is correlated with attitudes towards COVID-19. In the Netherlands, parents have the right to choose schools that match their values. A majority of schools are Christian (either Protestant, Catholic, Evangelic or Reformist), while around one third are public schools (Engzell et al., 2021). Other denominations include Islamic schools and Anthroposophic.

Students attended primary school together if they attended the same educational site (variable *WPOBRIN_crypt*) at the same branch (variable *WPOBRINVEST*, which allow us to distinguish between schools with different locations) and at the same year of education (variable *WPOLEER-JAAR*).

A.1.4. Registrations in secondary school (*Onderwijs-ONDERWIJSINSCHRTAB*) dataset

To assess if students attended secondary education (students aged 12+) together, we used the dataset *Onderwijs-ONDERWIJSINSCHRTAB*. This dataset contains all registrations in secondary schools. Similarly to the approach in primary schools, we kept only the school category (variable *TYPEONDERWIJS*) "VO" and excluded registrations in special schools. This dataset provides only registration of students by date of registration (variable *AANVINSCHR*) and deregistration (variable *EINDINSCHR*), not by year of study. Since we focus only on students in the first year of secondary school, registration date and year of study are expected to be almost identical—i.e, we assume students did not repeat year, which is highly unlikely for primary school. We kept students registering after August (and before September of the next year) in the same study year. We removed 1,884 students that were not registered for at least half a year in any school. This could happen for example if the student attended three or more schools in a year.

Students attended secondary school together if they attended the same educational site (variable *BRIN_crypt*), school branch (variable *VOBRINVEST*, which allow us to distinguish between schools with different locations), year of study derived from the date of registration (*AANVINSCHR*) as explained above, and program track of education (variable *OPLNR*), which allows us to distinguish students in the (pre-)vocational, general, applied, and scientific tracks. Students in different tracks attend different classrooms.

Bibliography

Engzell, P., Frey, A., & Verhagen, M. D. (2021). Learning loss due to school closures during the COVID-19 pandemic. *Proceedings of the National Academy of Sciences, 118*(17), e2022376118.
